# Prediction of Male Coronary Artery Bypass Grafting Outcomes Using Body Surface Area Weighted Left Ventricular End-diastolic Diameter: Multicenter Retrospective Cohort Study

**DOI:** 10.2196/45898

**Published:** 2023-03-23

**Authors:** Zhihui Zhu, Yuehuan Li, Fan Zhang, Stefanie Steiger, Cheng Guo, Nan Liu, Jiakai Lu, Guangpu Fan, Wenbo Wu, Mingying Wu, Huaibin Wang, Dong Xu, Yu Chen, Junming Zhu, Xu Meng, Xiaotong Hou, Hans-Joachim Anders, Jian Ye, Zhe Zheng, Chenyu Li, Haibo Zhang

**Affiliations:** 1 Department of Cardiac Surgery, Beijing Anzhen Hospital Capital Medical University Beijing China; 2 Medizinische Klinik und Poliklinik IV Hospital of the Ludwig-Maximilians-University Munich Germany; 3 Allianz Technology, Allianz Munich Germany; 4 Department of Cardiovascular Surgery, Peking University People's Hospital Beijing China; 5 Department of Cardiovascular Surgery, Beijing Tongren Hospital Capital Medical University Beijing China; 6 Department of Cardiovascular Surgery, Beijing Hospital Beijing China; 7 Department of Cardiovascular Surgery, Beijing Xuanwu Hospital Capital Medical University Beijing China; 8 Department of Cardiovascular Surgery, St. Paul's Hospital University of British Columbia Vancouver, BC Canada; 9 Department of Cardiovascular Surgery, Fuwai Hospital Chinese Academy of Medical Sciences & Peking Union Medical College Beijing China

**Keywords:** body surface area, BSA, left ventricular end-diastolic diameter, LVEDD, coronary artery bypass grafting, CABG, outcomes

## Abstract

**Background:**

The presence of a high left ventricular end-diastolic diameter (LVEDD) has been linked to a less favorable outcome in patients undergoing coronary artery bypass grafting (CABG) procedures. However, by taking into consideration the reference of left ventricular size and volume measurements relative to the patient's body surface area (BSA), it has been suggested that the accuracy of the predicting outcomes may be improved.

**Objective:**

We propose that BSA weighted LVEDD (bLVEDD) is a more accurate predictor of outcomes in patients undergoing CABG compared to simply using LVEDD alone.

**Methods:**

This study was a comprehensive retrospective cohort study that was conducted across multiple medical centers. The inclusion criteria for this study were patients who were admitted for treatment between October 2016 and May 2021. Only elective surgery patients were included in the study, while those undergoing emergency surgery were not considered. All participants in the study received standard care, and their clinical data were collected through the institutional registry in accordance with the guidelines set forth by the Society of Thoracic Surgeons National Adult Cardiac Database. bLVEDD was defined as LVEDD divided by BSA. The primary outcome was in-hospital all-cause mortality (30 days), and the secondary outcomes were postoperative severe adverse events, including use of extracorporeal membrane oxygenation, multiorgan failure, use of intra-aortic balloon pump, postoperative stroke, and postoperative myocardial infarction.

**Results:**

In total, 9474 patients from 5 centers under the Chinese Cardiac Surgery Registry were eligible for analysis. We found that a high LVEDD was a negative factor for male patients’ mortality (odds ratio 1.44, *P*<.001) and secondary outcomes. For female patients, LVEDD was associated with secondary outcomes but did not reach statistical differences for morality. bLVEDD showed a strong association with postsurgery mortality (odds ratio 2.70, *P*<.001), and secondary outcomes changed in parallel with bLVEDD in male patients. However, bLVEDD did not reach statistical differences when fitting either mortality or severer outcomes in female patients. In male patients, the categorical bLVEDD showed high power to predict mortality (area under the curve [AUC] 0.71, *P*<.001) while BSA (AUC 0.62) and LVEDD (AUC 0.64) both contributed to the risk of mortality but were not as significant as bLVEDD (*P*<.001).

**Conclusions:**

bLVEDD is an important predictor for male mortality in CABG, removing the bias of BSA and showing a strong capability to accurately predict mortality outcomes.

**Trial Registration:**

ClinicalTrials.gov NCT02400125; https://clinicaltrials.gov/ct2/show/NCT02400125

## Introduction

Coronary artery disease causes angina pectoris, myocardial infarction, and ischemic heart failure and thereby contributes significantly to the cardiovascular disease being the leading cause of death worldwide [1-3]. Coronary artery bypass graft (CABG) surgery is the gold-standard treatment in many patients with complex multivessel coronary artery disease or left main disease [4,5]. Left ventricular enlargement is a powerful predictor of adverse outcomes such as all-cause death, cardiovascular death, heart failure hospitalization, and outcomes of cardiac surgery [6-9]. For those undergoing CABG, enlarged left ventricular end-diastolic diameter (LVEDD) is most commonly associated with ischemic cardiomyopathy and is known to increase the risk of postoperative adverse events [10,11]. A more detailed study on the effects of LVEDD is needed to add more evidence about the relationship between the LVEDD and perioperative prognosis in CABG.

Body surface area (BSA) is a simple calculation based on the patients’ height and weight [12]. In contemporary cardiovascular care, BSA is used to normalize cardiac output to cardiac index, calculate glomerular filtration rate, is positively associated with blood pressure, and has been shown to be a relatively accurate representation of total body water [13-16]. The American Society of Echocardiography and the European Association of Cardiovascular Imaging have recommended using BSA to normalize echocardiographic parameters such as right ventricle size and left ventricle size [17]. Here, we propose to use the BSA to normalize the end-diastolic volume and to remove the bias of the BSA on LVEDD; thus, the BSA weighted LVEDD (bLVEDD) is defined as LVEDD divided by BSA. To this end, we intended to investigate the role of LVEDD, BSA, and bLVEDD in a specific clinical setting, and to evaluate whether the relationship among them after CABG will facilitate patient-specific care in cardiac surgery.

Hence, this study investigated the respective effect of LVEDD, BSA, and bLVEDD on early clinical outcomes in patients undergoing CABG by using clinical data from 5 top cardiac centers in China (Beijing Anzhen Hospital, Beijing Tongren Hospital, Beijing Hospital, Peking University People’s Hospital, and Beijing Xuanwu Hospital) under the Chinese Cardiac Surgery Registry database, to reveal (1) the effects of LVEDD on the perioperative prognosis, (2) the relationship between BSA and LVEDD, and (3) whether bLVEDD was associated with perioperative complications and mortality in patients undergoing CABG.

## Methods

### Study Setting and Population

This study was a multicenter retrospective analysis of observational data. A total of 9474 inpatients across the nation (Multimedia Appendix 1) from 5 top cardiac centers in China under the Chinese Cardiac Surgery Registry database, admitted between October 2016 and May 2021, were included in this study. All patients were elective surgery patients, and emergency surgery patients were excluded. Clinical data were obtained via the institutional registry following the Society of Thoracic Surgeons National Adult Cardiac Database. The accuracy and completeness of these data were ensured through multiple procedures, which have been described previously [18].

### Ethical Considerations

The study protocol had been approved by the Ethics Committee of Fuwai Hospital (Approval No. 2017-943). The study is registered at ClinicalTrials.gov (NCT02400125). To protect patient privacy, all patient data were deidentified (ie, patient names were replaced with the identification code, and all private patient information was deleted before analysis). The Peking University Clinical Research Institute has created a data committee to evaluate the data quality and supervise data collection. All patients were treated with standard care, and no additional intervention was performed as described previously [18].

### Predictor and Outcomes

Patient demographics and clinical characteristics were collected and analyzed. This included the patient’s past cardiovascular medical history (peripheral vascular disease, previous cerebrovascular event, previous myocardial infarction [MI], and previous percutaneous coronary intervention and New York Heart Association classification. The last preoperative test results of serum creatinine, total serum cholesterol, serum low-density lipoprotein, blood glucose, and estimated glomerular filtration rate (eGFR) were acquired. The patient’s previous echocardiogram before surgery was also analyzed for LVEDD and left atrial dimension. Intraoperative factors, such as cardiopulmonary bypass time, and aortic cross-clamp time were also analyzed. Variables for concomitant cardiac drugs (ie, nitrate lipid drugs, catecholamines, β-blockers, angiotensin-converting enzyme inhibitor, angiotensin receptor blocker, statins, aspirin, clopidogrel, and ticagrelor) were documented as comprehensively as possible. The primary outcome was in-hospital all-cause mortality (30 days). The secondary outcomes were postoperative severe adverse events, including use of extracorporeal membrane oxygenation, multiorgan failure, use of intra-aortic balloon pump, postoperative stroke, and postoperative MI. The BSA is calculated as follows [12]:



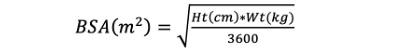



### Statistical Analysis

Variables with missing values or outliers warranted interpolation by multiple imputations using the MICE package [19]. Since the database was structurally designed and supervised by data committee, the missing values or outliers were less than 2% across all the indicators. We assumed that the data were missing or misrecording at random [20]; therefore, we performed predictive mean matching [21] to generate 5 complete imputed data sets that fit the logistic models. For multivariate logistic regression, we selected age, gender, smoking within 2 weeks before surgery, diabetes, hypertension, hyperlipidemia, last test of serum creatinine, total cholesterol, low-density lipoprotein, blood glucose, preoperative eGFR before surgery, and previous cerebrovascular events for adjustment based on clinical experience. The bLVEDD was optimally binned based on the weight of evidence binning by supervised tree-like segmentation; the process of generating the bin and threshold of the bLVEDD was follow by the reference pipeline of scorecard package [22]. The coefficient was calculated by Spearman correlation method. Categorical variables were compared using the chi-square test or Fisher exact test. Cochran-Armitage trend test was used for trend analysis. Continuous variables were compared using a 2-tailed *t* test or the Mann-Whitney *U* test. The area under the curve (AUC) of the receiver operating characteristics was compared using the DeLong method. The sample size calculation showed that an estimated 639 patients with a bLVEDD of ≥31.5 would be needed to provide 99% power for detecting a minimum clinically meaningful mortality rate of 5.23% with a 2-side α of .05 when compared with patients with a bLVEDD of <31.5. All analyses were performed using R version 3.4.2 (The R Project for Statistical Computing).

## Results

### Baseline

In total, 9474 patients were eligible for the final analysis, of which 7232 (76.34%) were male and 2242 (23.66%) were female. Among female patients, the mean age was 65.26 (SD 7.49) years, and 1032 (46.03%) had an LVEDD of <46 mm. In the male patients, mean age was 61.78 (SD 9.00) years, and 3615 (49.99%) had an LVEDD of <50 mm. Moreover, male patients with an LVEDD of <50 mm had a higher rate of smoking and comorbidity of hyperlipidemia, abnormal serum total cholesterol, and creatinine; a higher risk of previous MI; and a higher score of New York Heart Association (*P*<.05). Female patients with an LVEDD of <46 mm had more hypertension and previous MI and abnormal serum total cholesterol (*P*<.05). Both male and female patients with higher LVEDD had low left ventricular ejection fraction and left atrial dimension. Therefore, the preoperative baseline condition of patients with high LVEDD was worse than that of patients with low LVEDD (Table 1).

**Table 1:** Patient characteristics according to LVEDD^a^ category.

Characteristics			Gender															
			Female									Male						
			Total		LVEDD<46		LVEDD≥46		*P* value		Total			LVEDD<50		LVEDD≥50		*P* value
Total, n			2242		1032		1210		N/A^b^		7232			3615		3617		N/A
Age (years), mean (SD)			65.26 (7.49)		65.29 (7.6)		65.24 (7.39)		.87		61.78 (9.00)			62.12 (8.74)		61.43 (9.23)		.01
BMI (kg/m^2^), mean (SD)			25.34 (3.41)		24.76 (3.26)		25.82 (3.47)		<.001		25.77 (3.06			25.38 (3.04)		26..05 (3.05)		<.001
BSA^c^, mean (SD)			1.66 (0.14)		1.63 (0.13)		1.68 (0.14)		<.001		1.87 (0.14)			1.85 (0.14)		1.89 (0.14)		<.001
Smoking^d^, n (%)			193 (8.61)		85 (8.24)		108 (8.93)		.56		4075 (56.35)			1953 (54.02)		2122 (58.67)		<.001
Diabetes, n (%)			1027 (45.81)		473 (45.83)		554 (45.79)		.98		2708 (37.44)			1317 (36.43)		1391 (38.46)		.08
Hypertension, n (%)			1604 (71.54)		713 (69.09)		891 (73.64)		.02		4401 (60.85)			2189 (60.55)		2212 (61.16)		.60
Hyperlipidemia, n (%)			762 (33.99)		347 (33.62)		415 (34.3)		.74		2457 (33.97)			1289 (35.66)		1168 (32.29)		.003
Peripheral vascular disease, n (%)			73 (3.26)		32 (3.1)		41 (3.39)		.70		252 (3.48)			120 (3.32)		132 (3.65)		.44
Previous cerebrovascular event, n (%)			303 (13.51)		139 (13.47)		164 (13.55)		.95		986 (13.63)			500 (13.83)		486 (13.44)		.62
Previous MI^e^, n (%)			311 (13.87)		134 (12.98)		177 (14.63)		<.001		1331 (18.40)			503 (12.81)		828 (22.89)		<.001
Previous PCI^f^, n (%)			270 (12.04)		119 (11.53)		151 (12.48)		.49		1021 (14.12)			485 (13.42)		536 (14.82)		.86
**NYHA^g^, n (%)**																		
	NYHA1	1801 (80.33)		831 (80.52)		970 (80.17)		.29		5595 (77.36)			2717 (75.16)		2878 (79.57)		<.001	
	NYHA2	1311 (58.47)		623 (60.37)		688 (56.86)		—^h^		4084 (56.47)			2015 (55.74)		2069 (57.2)		—	
	NYHA3	459 (20.47)		195 (18.9)		264 (21.82)		—		1423 (19.68)			677 (18.73)		746 (20.62)		—	
	NYHA4	31 (1.38)		13 (1.26)		18 (1.49)		—		88 (1.22)			25 (0.69)		63 (1.74)		—	
Serum creatinine (umol/L)^i^, mean (SD)			63.58 (20.44)		63.21 (21.18)		63.89 (19.79)		.43		78.02 (22.23)			76.77 (20.19)		79.26 (24.04)		<.001
Serum total cholesterol (mmol/L), mean (SD)			4.23 (1.04)		4.28 (1.02)		4.19 (1.05)		.04		3.92 (0.97)			3.95 (0.97)		3.9 (0.97)		.03
Serum low-density lipoprotein, mean (SD)			2.51 (0.86)		2.53 (0.85)		2.49 (0.86)		.19		2.34 (0.81)			2.35 (0.81)		2.34 (0.81)		.74
eGFR^j^ (mL/min/1.73m^2^), mean (SD)			99.91 (11.76)		100.14 (11.7)		99.71 (11.81)		.38		93.88 (10.95)			94.08 (10.5)		93.69 (11.39)		.12
Blood glucose (mmol/L), mean (SD)			6.65 (2.02)		6.57 (1.96)		6.72 (2.08)		.08		6.44 (2.09)			6.4 (1.92)		6.48 (2.25)		.11
LVEF^k^, mean (SD)			61.65 (8.25)		63.34 (6.81)		60.22 (9.07)		<.001		59.31 (9.08)			62.38 (6.6)		56.24 (10.13)		<.001
LAD^l^ (mm), mean (SD)			34.83 (7.94)		34 (7.18)		35.53 (8.48)		<.001		36.52 (7.78)			35.4 (6.92)		37.63 (8.4)		<.001
LVEDD (mm), mean (SD)			46.32 (4.96)		42.28 (2.55)		49.75 (3.78)		<.001		49.99 (5.9)			45.46 (3.09)		54.52 (4.38)		<.001
Normalized by weight, mean (SD)			0.75 (0.12)		0.7 (0.11)		0.78 (0.13)		<.001		0.68v(0.12)			0.63 (0.1)		0.73 (0.11)		<.001
Normalized by BMI, mean (SD)			1.86 (0.3)		1.74 (0.25)		1.96 (0.3)		<.001		1.97 (0.32)			1.79 (0.24)		2.09 (0.3)		<.001
Normalized by BSA, mean (SD)			28.05 (3.43)		26.09 (2.49)		29.73 (3.23)		<.001		26.85 (3.54)			24.72 (2.45)		28.99 (3.15)		<.001
Nitrate lipid drugs^m^, n (%)			547 (24.4)		232 (22.48)		315 (26.03)		.51		1696 (23.45)			787 (21.77)		909 (25.13)		<.001
Catecholamines^n^, n (%)			15 (0.67)		6 (0.58)		9 (0.74)		.64		33 (0.46)			17 (0.47)		16 (0.44)		.86
β-blockers^o^, n (%)			1860 (82.96)		866 (83.91)		994 (82.15)		.27		6011 (83.12)			2974 (82.27)		3037 (83.96)		.05
ACEI^p^ or ARB^q,r^, n (%)			539 (24.04)		230 (22.29)		309 (25.54)		.07		1489 (20.59)			704 (19.47)		785 (21.7)		.02
Statins, n (%)			1519 (67.75)		708 (68.6)		811 (67.02)		.42		4942 (68.34)			2460 (68.05)		2482 (68.62)		.60
Aspirin^s^, n (%)			672 (29.97)		295 (28.59)		377 (31.16)		.19		2234 (30.89)			1016 (28.11)		1218 (33.67)		<.001
Clopidogrel, n (%)			145 (6.47)		74 (7.17)		71 (5.87)		.21		547 (7.56)			276 (7.63)		271 (7.49)		.82
Ticagrelor, n (%)			86 (3.92)		46 (4.6)		40 (3.36)		.14		343 (4.84)			188 (5.37)		155 (4.33)		.04

^a^LVEDD: left ventricular end-diastolic diameter.

^b^N/A: not applicable.

^c^BSA: body surface area.

^d^Smoking within 2 weeks before surgery.

^e^MI: myocardial infarction.

^f^PCI: percutaneous coronary intervention.

^g^NYHA: New York Heart Association.

^h^Not available.

^i^Serum creatinine, serum total cholesterol, serum low-density lipoprotein, eGFR, blood glucose, LVEF, LVEDD, and LAD are the last tests before surgery.

^j^eGFR: estimated glomerular filtration rate.

^k^LVEF: left ventricular ejection fraction.

^l^LAD: left atrial dimension.

^m^Nitrate lipid drugs are administered intravenously 24 hours before surgery.

^n^Catecholamines are administered intravenously 48 hours before surgery.

^o^β-blockers and statins are administered orally 24 hours before surgery.

^p^ACEI: angiotensin-converting enzyme inhibitor.

^q^ARB: angiotensin receptor blocker.

^r^ACEI or ARB are administered orally 48 hours before surgery.

^s^Aspirin, clopidogrel, and ticagrelor are administered orally 5 days before surgery.

### High LVEDD is a Negative Prognostic Factor for Male Patients

**Figure 1:**
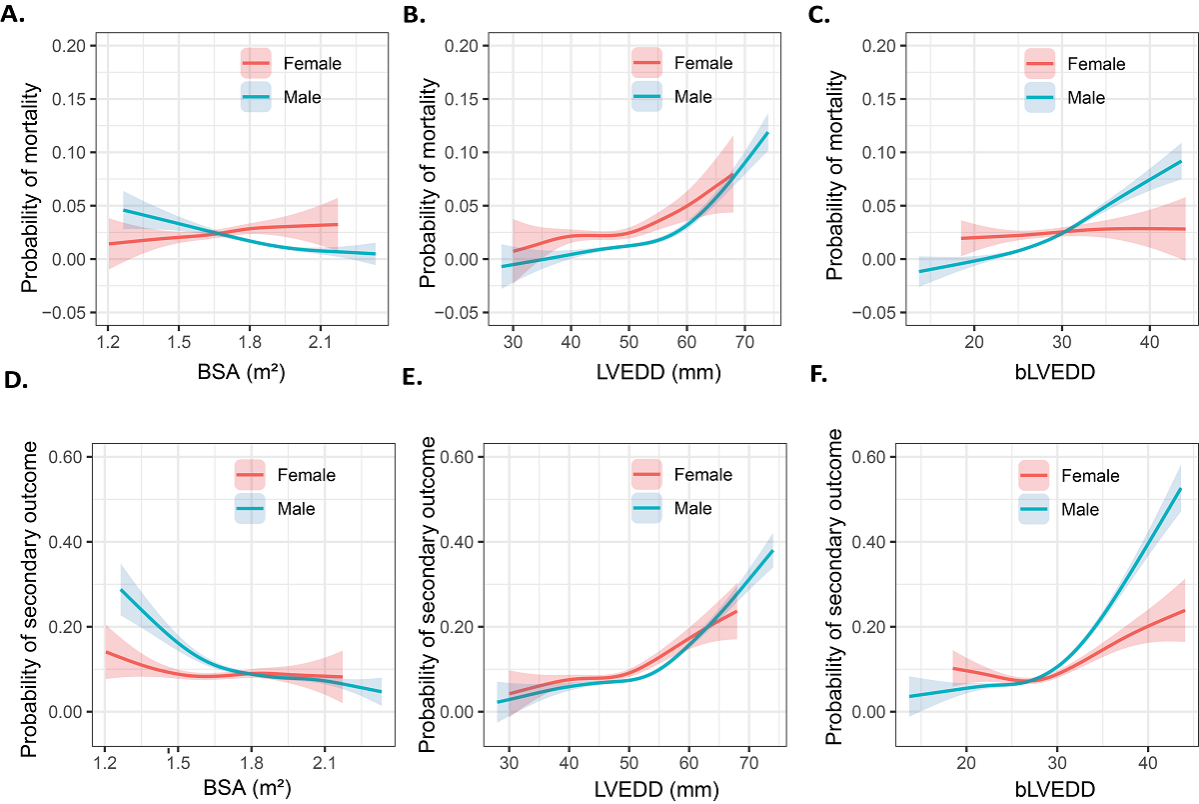
Generalized additive models of body surface area (BSA), left ventricular end-diastolic diameter (LVEDD), and body surface area weighted left ventricular end-diastolic diameter (bLVEDD) for primary and secondary outcomes. (A-C) Probability of mortality. (D-F) Secondary outcomes using restricted cubic splines.

As shown in Table 2, both male and female patients with high LVEDD yielded lower eGFR (*P*<.001). Male patients with high LVEDD had longer mechanical ventilation duration, initial intensive care unit length of stay, higher serum creatinine, more use of intra-aortic balloon pump and extracorporeal membrane oxygenation, and higher mortality (*P*<.001), while in female patients, these do not reach statistical difference (Table 2). As a regression result, high LVEDD was a negative factor for male patients’ mortality (adjusted odds ratio [OR] 1.44, 1.33-1.56, *P*<.001; Table 3, Figure 1B), indicating that each increase in the patient’s LVEDD fifths classification increased the odd of mortality by 44%. Similarly, male patients with high LVEDD had more secondary adverse events (adjusted OR 1.19, 1.16-1.23, *P*<.001; Table 3, Figure 1B) by increasing the odd by 16% for each increase in the LVEDD classification. For female patients, the LVEDD was associated with secondary outcomes (OR 1.13, 1.07~1.19, *P*=.03), but did not reach statistical difference for morality (Multimedia Appendix 2). Therefore, high LVEDD is a negative prognostic factor for both postoperative survival and severe events in male patients.

**Table 2:** Patient outcomes according to left ventricular end-diastolic diameter (LVEDD) category.

Characteristics	Gender								
	Female					Male			
	Total	LVEDD<46	LVEDD≥46	*P* value	Total		LVEDD<50	LVEDD≥50	*P* value
Perioperative blood transfusion, n (%)	1672 (74.58)	763 (73.93)	909 (75.12)	.52	4740 (65.54)		2393 (66.2)	2347 (64.89)	.24
Mechanical ventilation duration (hour), mean (SD)	26.22 (29.58)	25.57 (28.2)	26.77 (30.7)	.34	24.43 (26.9)		23.05 (23.29)	25.8 (30.03)	<.001
Initial ICU^a^ length of stay (hour), mean (SD)	37.79 (39.99)	36.5 (38.19)	38.89 (41.45)	.16	36.45 (39.83)		32.9 (33.39)	39.99 (45.08)	<.001
Perioperative blood loss (ml), mean (SD)	915.1 (809.39)	899.53 (809.24)	928.38 (809.62)	.40	1087.39 (900.03)		1081.72 (929.07)	1093.05 (870.12)	.59
Serum creatinine^b^ (umol/L), mean (SD)	78.45 (43.49)	77.08 (45.72)	79.62 (41.48)	.17	91.7 (40.37)		89.9 (38.2)	93.51 (42.37)	<.001
eGFR^c^ (mL/min/1.73m2), mean (SD)	105.15 (34.46)	107.53 (34.24)	103.12 (34.53)	.003	87.6 (28.3)		88.64 (27.82)	86.55 (28.74)	.002
AKI^d^, n (%)	313 (13.96)	136 (13.18)	177 (14.63)	.32	715 (9.89)		348 (9.63)	367 (10.15)	.46
Use of IAPB^e^, n (%)	155 (6.91)	65 (6.3)	90 (7.44)	.29	522 (7.22)		208 (5.75)	314 (8.68)	<.001
Use of ECMO^f^, n (%)	18 (0.8)	9 (0.87)	9 (0.74)	.73	53 (0.73)		28 (0.77)	25 (0.69)	.67
Multiorgan failure, n (%)	31 (1.38)	12 (1.16)	19 (1.57)	.41	64 (0.88)		25 (0.69)	39 (1.08)	.08
Reoperation, n (%)	49 (2.19)	24 (2.33)	25 (2.07)	.90	151 (2.09)		73 (2.02)	78 (2.16)	.58
Postoperative MI^g^, n (%)	10 (0.45)	4 (0.39)	6 (0.5)	.70	64 (0.88)		29 (0.8)	35 (0.97)	.45
Postoperative stroke, n (%)	33 (1.47)	14 (1.36)	19 (1.57)	.68	64 (0.88)		30 (0.83)	34 (0.94)	.62
Reintubation, n (%)	21 (0.94)	9 (0.87)	12 (0.99)	.77	77 (1.06)		22 (0.61)	55 (1.52)	<.001
Re-enter ICU, n (%)	45 (2.01)	16 (1.55)	29 (2.4)	.15	118 (1.63)		56 (1.55)	62 (1.71)	.58
Dead, n (%)	54 (2.41)	23 (2.23)	31 (2.56)	.61	107 (1.48)		33 (0.91)	74 (2.05)	<.001

^a^ICU: intensive care unit.

^b^Serum creatinine is the maximum serum creatinine after surgery.

^c^eGFR: estimated glomerular filtration rate. This is the minimum eGFR after surgery.

^d^AKI: acute kidney injury.

^e^IAPB: intra-aortic balloon pump.

^f^ECMO: extracorporeal membrane oxygenation.

^g^MI: myocardial infarction.

**Table 3:** Adjusted and unadjusted logistic regression model of the association between body surface area weighted left ventricular end-diastolic diameter (bLVEDD) and prognosis of male patients^a^.

Variables			Secondary outcomes												Mortality										
			Univariate				Multivariate					AUC^b^			Univariate					Multivariate					AUC
			OR^c^		*P* value		OR		*P* value					OR			*P* value		OR			*P* value			
**bLVEDD^d^**																									
	Numerical bLVEDD	1.13 (1.12~1.15)		<.001		1.12 (1.1~1.13)		<.001		0.61			1.21 (1.18~1.24)			<.001		1.18 (1.15~1.21)			<.001		0.71		
	Categorized bLVEDD	1.76 (1.66~1.86)		<.001		1.64 (1.55~1.73)		<.001		0.59			3.08 (2.69~3.53)			<.001		2.7 (2.36~3.1)			<.001		0.71		
	<22.5	0.73 (0.57~0.92)		.17		0.65 (0.51~0.82)		0.07		N/A^e^			0 (0~0)			.98		0 (0~0)			.98		N/A		
	(22.5, 26)	1		N/A		1		N/A		N/A			1			N/A		1			N/A		N/A		
	(26, 31.5)	1.39 (1.26~1.53)		<.001		1.32 (1.19~1.45)		<.001		N/A			2.7 (2.07~3.52)			<.001		2.44 (1.87~3.19)			<.001		N/A		
	(31.5, infinity)	3.6 (3.2~4.06)		<.001		3 (2.65~3.39)		<.001		N/A			8.63 (6.52~11.43)			<.001		6.54 (4.9~8.73)			<.001		N/A		
**LVEDD^f^**																									
	Numerical LVEDD	1.07 (1.06~1.07)		<.001		1.06 (1.05~1.07)		<.001		0.59			1.1 (1.09~1.12)			<.001		1.09 (1.08~1.11)			<.001		0.65		
	Categorized LVEDD	1.22 (1.18~1.26)		<.001		1.19 (1.16~1.23)		<.001		0.58			1.48 (1.36~1.6)			<.001		1.44 (1.33~1.56)			<.001		0.64		
	<45	1		N/A		1		N/A		N/A			1			N/A		1			N/A		N/A		
	(45, 48)	1.18 (1.01~1.39)		.30		1.32 (1.12~1.55)		.09		N/A			1.93 (1.19~3.13)			.18		2.33 (1.43~3.79)			.08		N/A		
	(48, 51)	1.17 (0.98~1.38)		.37		1.25 (1.05~1.49)		.2		N/A			2.03 (1.23~3.35)			.16		2.3 (1.39~3.8)			.10		N/A		
	(51, 54)	1.24 (1.07~1.44)		.15		1.37 (1.18~1.6)		.04		N/A			2.33 (1.47~3.68)			.07		2.85 (1.8~4.53)			.02		N/A		
	(54, infinity)	2.29 (1.99~2.64)		<.001		2.23 (1.93~2.58)		<.001		N/A			5.45 (3.53~8.41)			<.001		5.58 (3.61~8.64)			<.001		N/A		
**BSA^g^**																									
	Numerical BSA	0.3 (0.23~0.41)		<.001		0.36 (0.27~0.49)		<.001		0.54			0.07 (0.04~0.14)			<.001		0.11 (0.06~0.23)			<.001		0.61		
	Categorized BSA	0.88 (0.85~0.91)		<.001		0.9 (0.87~0.93)		.002		0.54			0.72 (0.67~0.77)			<.001		0.76 (0.7~0.82)			<.001		0.62		
	<1.75	1		N/A		1		N/A		N/A			1			N/A		1			N/A		N/A		
	(1.75, 1.83)	0.75 (0.64~0.87)		.06		0.75 (0.64~0.88)		.06		N/A			0.68 (0.5~0.92)			.20		0.7 (0.52~0.96)			.25		N/A		
	(1.83, 1.91)	0.67 (0.58~0.77)		<.001		0.68 (0.58~0.79)		.01		N/A			0.54 (0.4~0.73)			.04		0.57 (0.42~0.78)			.07		N/A		
	(1.91, 1.99)	0.6 (0.52~0.7)		<.001		0.63 (0.54~0.73)		.003		N/A			0.35 (0.25~0.48)			<.001		0.39 (0.28~0.55)			<.001		N/A		
	(1.99, infinity)	0.56 (0.48~0.65)		<.001		0.6 (0.52~0.71)		<.001		N/A			0.26 (0.19~0.37)			<.001		0.33 (0.23~0.47)			<.001		N/A		

^a^Age, gender, smoking within 2 weeks before surgery, diabetes, hypertension, hyperlipidemia, last test of serum creatinine before surgery, last test of serum total cholesterol before surgery, last test of serum low-density lipoprotein before surgery, last test of blood glucose before surgery, use of cardiopulmonary bypass, preoperative estimated glomerular filtration rate, and previous cerebrovascular events were used for the multivariate regression. bLVEDD was categorized into 4 groups based on a weight of tree-like segmentation binning.

^b^AUC: area under the curve.

^c^OR: odds ratio.

^d^bLVEDD is LVEDD divided by BSA.

^e^N/A: not applicable.

^f^LVEDD: left ventricular end-diastolic diameter.

^g^BSA: body surface area.

### LVEDD Normalized for BSA

In our cohort, the mean BSA was 1.66 m^2^ (SD 0.14 m^2^) for female and 1.87 m^2^ (SD 0.14 m^2^) for male patients, which showed a slightly positive relationship (*R*=0.20 for female and *R*=0.15 for male patients, *P*<.001; Multimedia Appendix 3) with the LVEDD and reached statistical difference when compared in high and low LVEDD groups (*P*<.001). In male patients, high BSA was significantly associated with mortality (adjusted OR 0.76, 0.70-0.82, *P*<.001; Table 3, Figure 1A) and secondary outcomes (adjusted OR 0.90, 0.87-0.93, *P*<.001; Table 3, Figure 1D). However, in female patients, the BSA was not associated with either mortality or secondary outcomes.

Since the LVEDD has not been analyzed together with BSA in patients in CABG previously, we speculated that bLVEDD, defined as LVEDD divided by BSA, could better predict postsurgery prognosis. To identify whether bLVEDD increases the risk of postoperative mortality and secondary outcomes as well as whether it is the better predictor of outcomes, both univariate and multivariate logistic regression analyses were performed. As a result, in male patients, bLVEDD showed a strong association with postsurgery mortality; that is, the risk of mortality (adjusted OR 2.70, 2.39-3.10, *P*<.001; Table 3, Figure 1E). Secondary outcomes (adjusted OR 1.64, 1.55-1.73, *P*<.001; Table 3, Figure 1F) changed in parallel with a rise in bLVEDD, suggesting that bLVEDD represents a predictor for mortality and secondary outcomes. However, the bLVEDD did not reach statistical difference when fitting either mortality or severer outcomes in female patients.

### The bLVEDD is a Robust Indicator for Mortality in Male Patients

**Figure 2:**
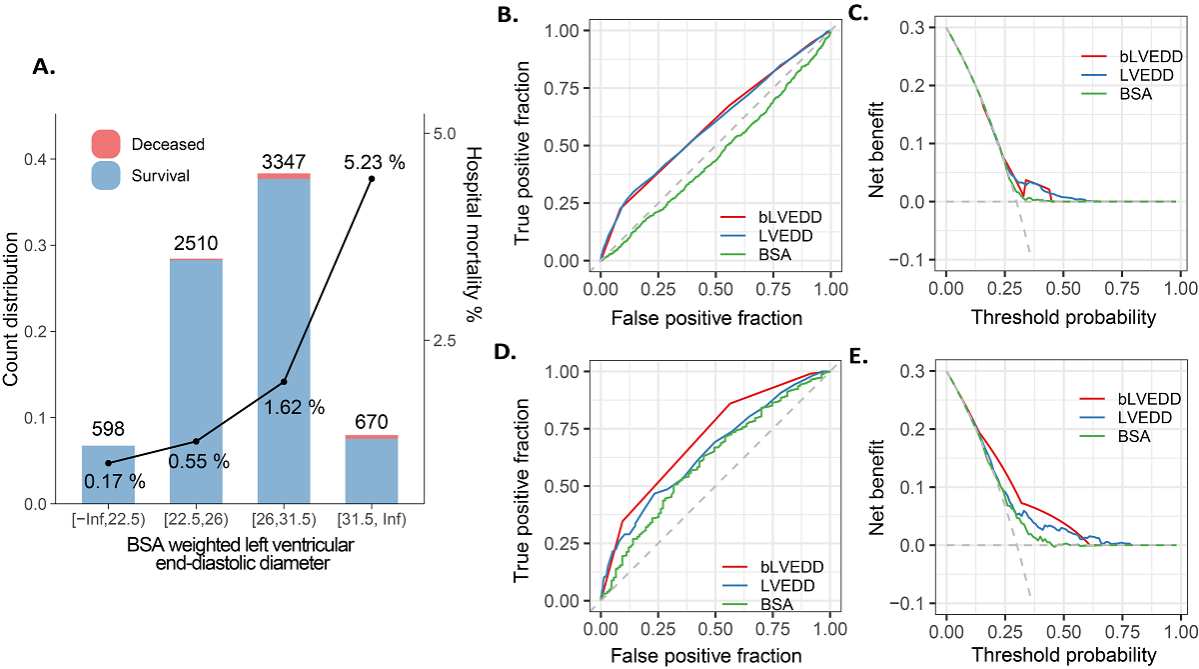
Segmentation of bLVEDD and its ability to predict clinical outcome in male patients. (A) Supervised tree-like segmentation of bLVEDD; (B) receiver operating characteristics; (C) precision-recall for secondary outcomes; and (D,E) motility. bLVEDD: body surface area weighted left ventricular end-diastolic diameter; BSA: body surface area; LVEDD: left ventricular end-diastolic diameter.

To make bLVEDD more practical for male patients undergoing CABG, a weight of tree-like segmentation was used to binning bLVEDD to a categorical variable. As a result, our data set generated a categorization of (0, 22.5), (22.5, 26), (26, 31.5), (31.5, infinity; adjusted Kolmogorov-Smirnov 0.38, *P*=.01; Figure 2A, Multimedia Appendix 4). The categorical bLVEDD showed a quite similar power to a numerical form to predict mortality (AUC 0.71, *P*<.001). However, compared with BSA and LVEDD, bLVEDD was the most effective variable that fitted with mortality. The compositions of bLVEDD, such as BSA (AUC 0.62) and LVEDD (AUC 0.64), all slightly contributed to the risk of mortality with low AUC (DeLong test, *P*<.001) but were not as significant as that of bLVEDD (Figure 2B-E). Importantly, male patients with a bLVEDD of <31.5 faced higher mortality risk than those with a bLVEDD of ≥31.5 (OR 5.09, 4.14~6.26, *P*<.001; Figure 2D and E).

## Discussion

### Principal Findings

In this multicenter cohort study, we reported the following: (1) a high LVEDD is a negative prognostic factor for both postoperative survival and secondary outcomes in male patients; (2) in male patients, high BSA was significantly associated with mortality and secondary outcomes, while female patients’ BSA is not associated with either mortality or secondary outcomes; (3) bLVEDD showed a strong association with postsurgery mortality; that is, the risk of mortality and secondary outcomes changed in parallel with bLVEDD increasing in male patients, but female patients’ bLVEDD did not reach statistical difference; (4) A bLVEDD of 31.5 is the threshold to categorize male patients undergoing CABG with a high or low risk of mortality.

It is known that severe left ventricular dysfunction is associated with increased mortality in patients undergoing CABG [23-25]. Left ventricular function was usually described as the ejection fraction (EF) [26-28]; however, it is unclear whether EF is the most meaningful index of left ventricular function in a CABG situation. The left ventricular ejection fraction is the fraction of the end-diastolic volume that is ejected with each beat; that is, stroke volume divided by end-diastolic volume. Low EF may be caused by poor contractile function duo to extensive myocardial damage, or infarct expansion and stretching of the myocardial scar [29]. Thus, LVEDD might be a more meaningful predictor than EF, which is merely an arithmetical term based on 2 values. Zhu et al [10] reported that left ventricular geometry was an independent and incremental prognostic factor for death in patients undergoing CABG. Yan et al [11] found that left ventricular hypertrophy and left ventricular enlargement were associated with an increased risk of postoperative mortality after CABG in patients with heart failure with reduced ejection fraction. Categorizing left ventricular structural patterns with left ventricular hypertrophy and left ventricular enlargement contributes to risk stratification and provides incremental predictive ability. In our study, we also found that patients with high LVEDD had a poor baseline and suffered from more comorbidities, and LVEDD is an adverse prognostic factor for both postoperative survival and secondary outcomes in male patients undergoing CABG, which is consistent with previous studies.

Echocardiography is widely used in the diagnosis of cardiac diseases, especially for patients undergoing cardiac surgery. The measurement of the size of the left ventricle should be a part of every echocardiography report, because it provides diagnostic clues and prognostic information and enables the clinician to follow up with patients in respect of disease progression [30,31]. In clinical practice, surgeons often evaluate echocardiographic indicators using unstandardized absolute values; however, the structural characteristics of the heart should be related to human body measures such as height and weight under normal physiological conditions. Simply evaluating the absolute value of the left ventricle is not conducive to an accurate diagnosis of cardiac disease [32]. BSA is a critical index of physiologic functions, and it is used in several medical disciplines, including cardiology, oncology, burn management, and nephrology [33]. Some studies reported that the left ventricular diameter is a relatively crude and simplified assessment of a 3D structure, which cannot consider more complex variations in ventricular shape or size [34]. Using BSA to normalize echocardiographic parameters has been recommended by guidelines [17]. In the recommendations for chamber quantification, orthogonal long-axis views and the Simpson biplane method allow a more accurate calculation of the left ventricular volume, which may be corrected for size by normalizing to BSA [35]. LVEDD is data within echocardiography and is seen when the ventricle is the largest, shortly before the mitral valve closes and the mitral annulus descends. LVEDD is recognized as a negative risk factor for CABG. However, the definition of the normal range of the LVEDD is based on the entire population, including groups with different clinical characteristics. In our study, we found that the larger the LVEDD, the higher the perioperative mortality, even in the normal range. This may lead to misjudgment by cardiac surgeons, who believe that heart function is relatively safe when CABG is performed in patients with a “normal” LVEDD. Therefore, the risk factor of the left ventricle should be considered comprehensively when predicting the perioperative outcomes of CABG, and LVEDD should be normalized to remove the bias. We used BSA normalized LVEDD more accurately to predict mortality in patients undergoing CABG.

There is evidence to suggest gender inequality in CABG [36]. Studies have shown that female patients are at a higher risk of short-term mortality and other complications after surgery [37,38]. In our study, we also found that female patients have higher mortality compared to male patients undergoing CABG (2.41% vs 1.48%, *P*<.001). Some studies showed that the type of diffuse coronary disease is more commonly seen in female patients, which may be a contributing factor to poor outcomes [39,40]. Furthermore, some cardiovascular risk factors, such as diabetes and smoking, have a severer influence among female patients [39]. It is suggested that the baseline characteristics and unequal risk factors are the reasons for the difference in the outcomes after CABG [36]. For the LVEDD, guidelines also suggested that female and male patients have different normal ranges [17]. In our results, the bLVEDD also showed a disparity between different genders. In male patients undergoing CABG, bLVEDD showed a strong association with postsurgery mortality, while in female patients, the bLVEDD did not reach statistical difference when fitting either mortality or severer outcomes. Therefore, the gender disparity observed in bLVEDD should also be further studied in larger cohorts.

### Limitations

There are some limitations in this study. First, our study is a retrospective cohort study. The data collection of patients in the past is limited, the preoperative activity tolerance of patients is difficult to obtain, the follow-up time was long, and the rate of loss to follow-up was high. Second, intractable heart failure and atrial fibrillation are also common complications after CABG, but they were not included in this study because they were difficult to record accurately during follow-up. Third, BSA is an empirical formula based on weight and height and cannot directly give the true numerical value of the human surface. Especially in this context, BSA is confounded with age, gender, race, etc; thus, a further study is needed to study the factors that have collinearity with BSA.

### Conclusions

The bLVEDD is an important predictor for male mortality in CABG, removing the bias of BSA and showing a strong capability to accurately predict mortality outcomes. In predicting perioperative outcomes of CABG, it is important to comprehensively consider the risk factor of left ventricular enlargement and normalize LVEDD by BSA to eliminate bias in male patients. This research highlights significant benefits for enhancing the treatment standards of cardiac surgery and increasing the survival rate of patients following CABG.

## Appendix

Multimedia Appendix 1 Regional distribution of enrolled patients.

Multimedia Appendix 2 Adjusted and unadjusted logistic regression model of the association between body surface area weighted left ventricular end-diastolic diameter (bLVEDD) and prognosis of female patients.

Multimedia Appendix 3 Scatter plot and density distribution of left ventricular end-diastolic diameter (LVEDD) and body surface area (BSA).

Multimedia Appendix 4 Supervised tree-like segmentation of bLVEDD and its evaluation and validation. AUC: Area Under the Curve; F-1: F1 Score; FPR: False Positive Rate; K-S: Kolmogorov-Smirnov; P-R: Precision-Recall; ROC: Receiver Operating Characteristic; TPR: True Positive Rate.

## Abbreviations

AUC: area under the curve
bLVEDD: body surface area weighted left ventricular end-diastolic diameter
BSA: body surface area
CABG: coronary artery bypass grafting
EF: ejection fraction
eGFR: estimated glomerular filtration rate
LVEDD: left ventricular end-diastolic diameter
MI: myocardial infarction
OR: odds ratio

## Data Availability

The data sets generated or analyzed during this study are available from the corresponding author on reasonable request.
