# Supplementary figures and images for: Prediction of Male Coronary Artery Bypass Grafting Outcomes Using Body Surface Area Weighted Left Ventricular End-diastolic Diameter: Multicenter Retrospective Cohort Study
Source: Interact J Med Res. 2023 Mar 23;12:e45898. doi: 10.2196/45898 (PMC10131828; doi:10.2196/45898)

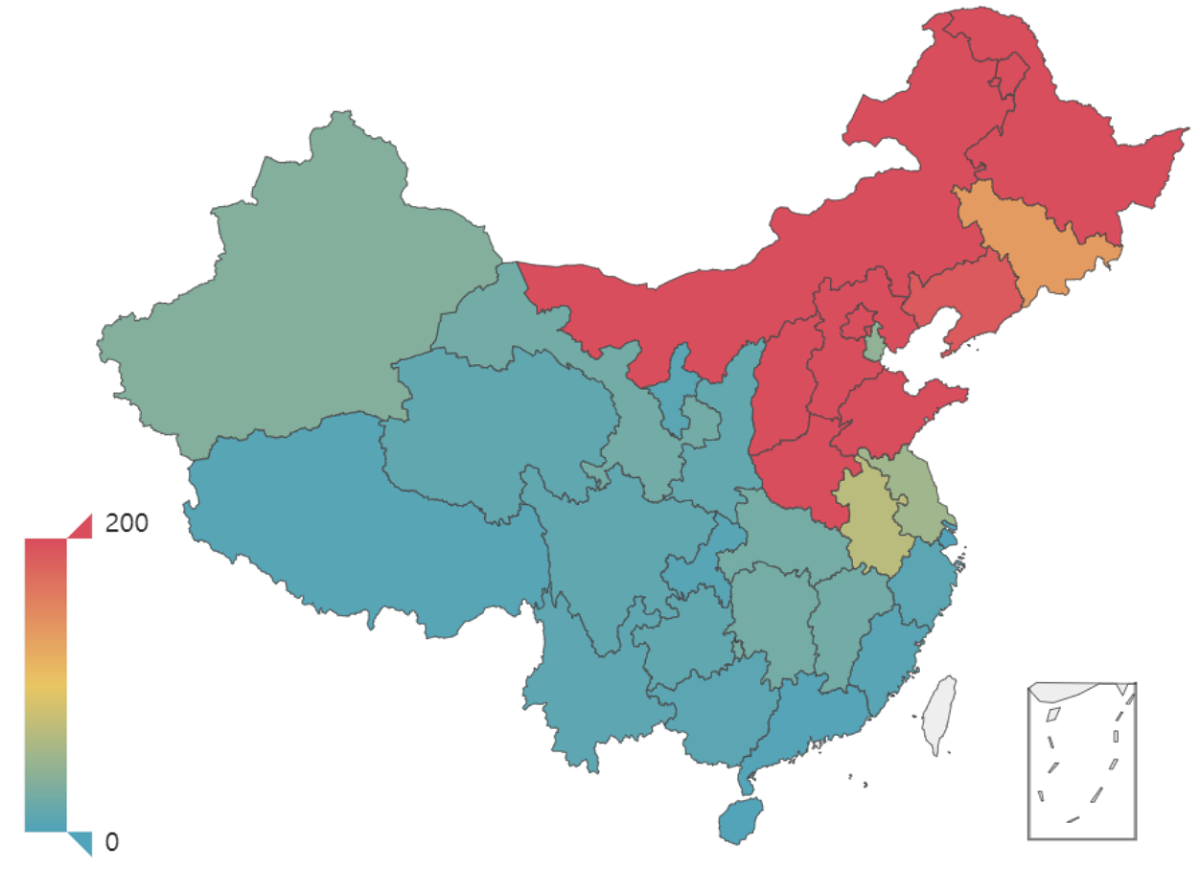

Supplement: Multimedia Appendix 1 [file ijmr_v12i1e45898_app1.png]

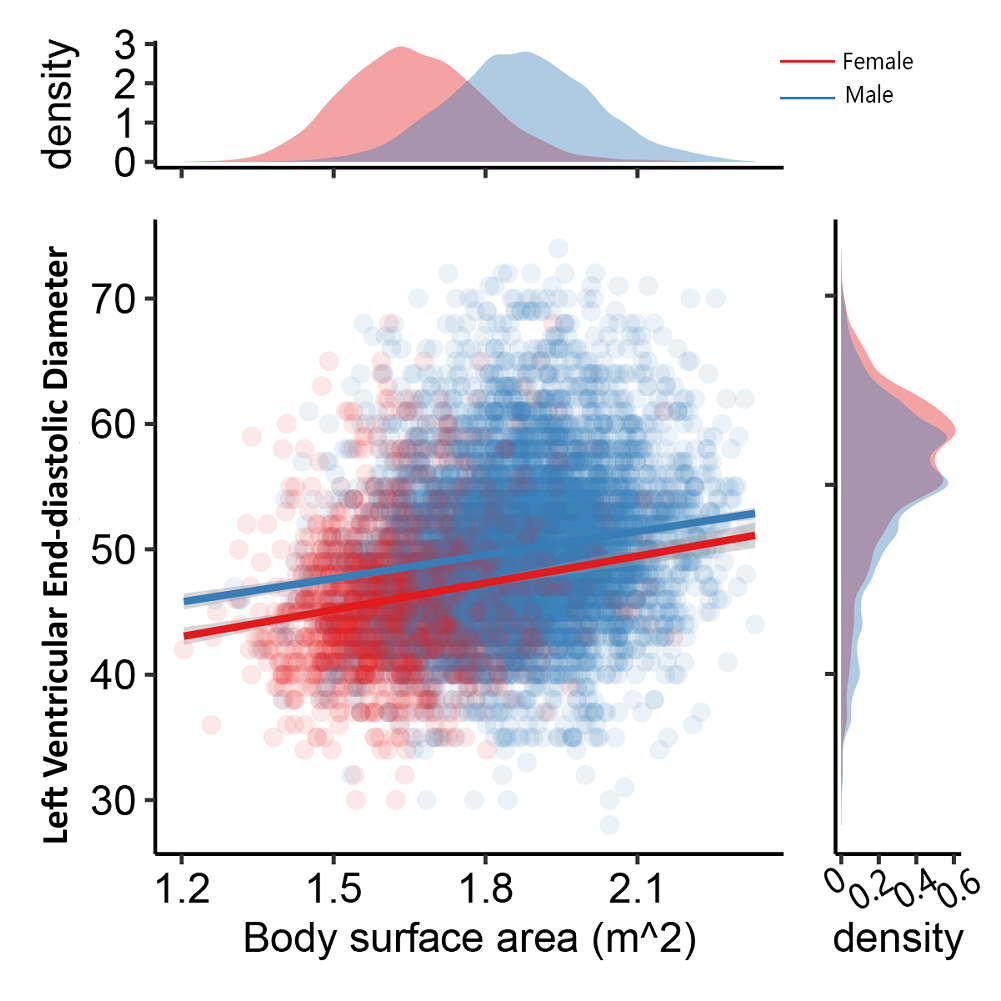

Supplement: Multimedia Appendix 3 [file ijmr_v12i1e45898_app3.png]

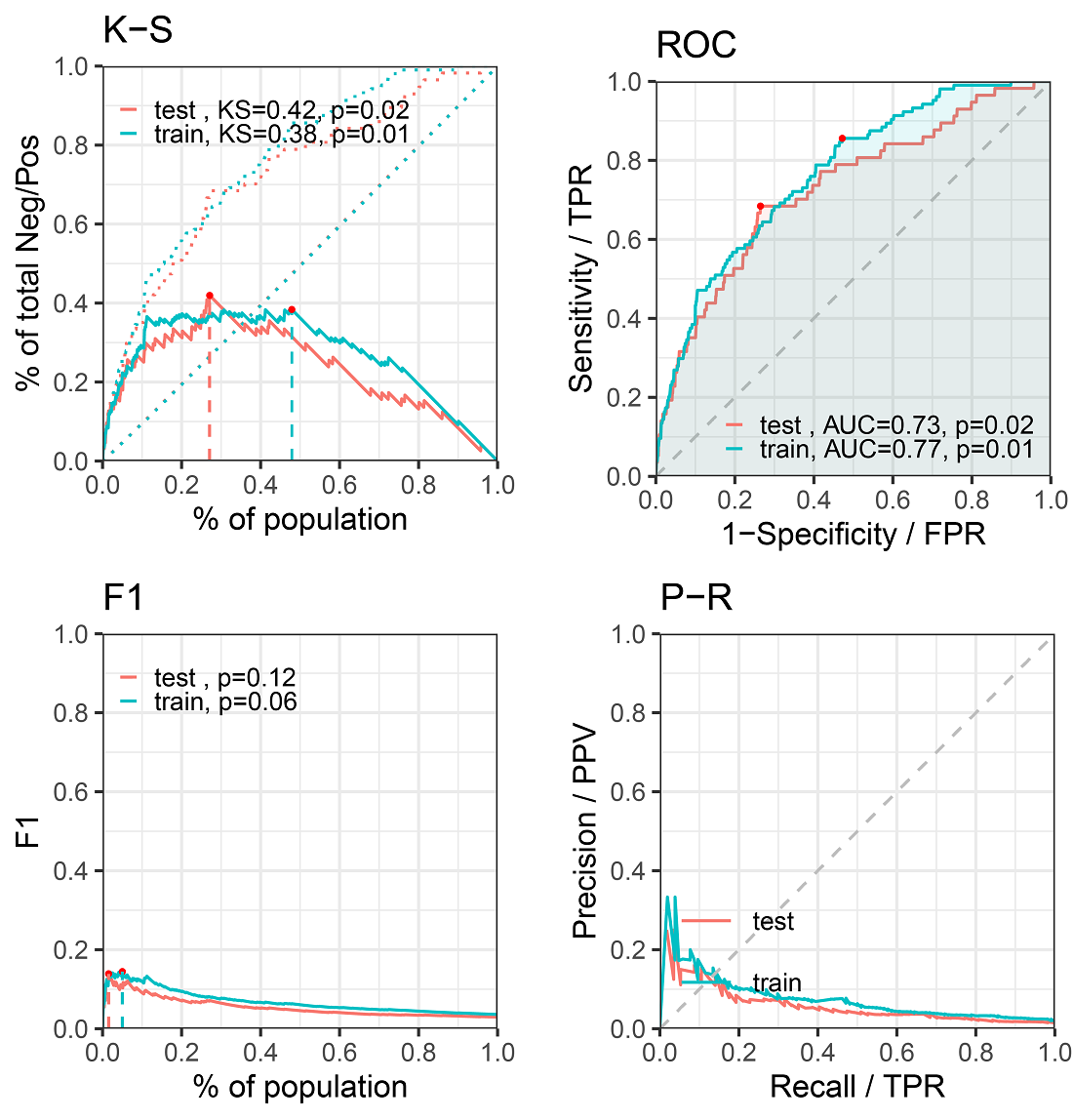

Supplement: Multimedia Appendix 4 [file ijmr_v12i1e45898_app4.png]
