# Supplementary material for: Prediction of Male Coronary Artery Bypass Grafting Outcomes Using Body Surface Area Weighted Left Ventricular End-diastolic Diameter: Multicenter Retrospective Cohort Study
Source: Interact J Med Res. 2023 Mar 23;12:e45898. doi: 10.2196/45898 (PMC10131828; doi:10.2196/45898)
Supplement: Multimedia Appendix 2 [file ijmr_v12i1e45898_app2.docx]

**Multimedia Appendix 2: Adjusted and unadjusted Logistic Regression Model of the association between bLVEDD and prognosis of Female patients***

|  | **Secondary outcomes** | | | | |  | **Mortality** | | | | |
| --- | --- | --- | --- | --- | --- | --- | --- | --- | --- | --- | --- |
|  | **Univariate** | | **Multivariate** | | **AUC** |  | **Univariate** | | **Multivariate** | | **AUC** |
|  | **OR** | ***P* value** | **OR** | ***P* value** |  |  | **OR** | ***P* value** | **OR** | ***P* value** |  |
| Numerical bLVEDD | 1.06(1.04~1.09) | <.001 | 1.06(1.03~1.08) | <.001 | 0.55 |  | 1.02(0.98~1.06) | .55 | 1.02(0.98~1.06) | .67 | 0.53 |
| Categorized bLVEDD | 1.25(1.13~1.39) | .03 | 1.21(1.09~1.35) | .07 | 0.53 |  | 1.19(0.98~1.45) | .37 | 1.14(0.94~1.38) | .51 | 0.53 |
| <22.5 | 1.75(1.14~2.68) | .19 | 1.54(1~2.39) | .32 |  |  | 0.86(0.3~2.44) | .89 | 0.62(0.22~1.8) | .66 |  |
| [22.5,26.5) | 1 |  | 1 |  |  |  | 1 |  | 1 |  |  |
| [26.5,31.5) | 0.94(0.78~1.12) | .71 | 0.93(0.78~1.12) | .70 |  |  | 1.13(0.81~1.56) | .71 | 1.07(0.77~1.49) | .83 |  |
| [31.5, INF) | 1.99(1.61~2.46) | <.001 | 1.83(1.47~2.27) | <.001 |  |  | 1.45(0.96~2.2) | .37 | 1.23(0.8~1.89) | .63 |  |
|  |  |  |  |  |  |  |  |  |  |  |  |
| Numerical LVEDD | 1.04(1.03~1.06) | <.001 | 1.04(1.03~1.06) | <.001 | 0.55 |  | 1.04(1.02~1.07) | .11 | 1.04(1.01~1.07) | .14 | 0.54 |
| Categorized LVEDD | 1.16(1.09~1.22) | <.001 | 1.13(1.07~1.19) | .03 | 0.54 |  | 1.15(1.03~1.27) | .18 | 1.11(1~1.23) | .30 | 0.54 |
| <42 | 1 |  | 1 |  |  |  | 1 |  | 1 |  |  |
| [42,45) | 1.09(0.9~1.33) | .65 | 1.15(0.94~1.4) | .48 |  |  | 1.04(0.72~1.49) | .92 | 1.12(0.78~1.62) | .76 |  |
| [45,57) | 0.76(0.59~0.97) | .27 | 0.79(0.61~1.02) | .35 |  |  | 0.95(0.62~1.47) | .91 | 1.03(0.66~1.6) | .96 |  |
| [47,5.) | 1.2(0.94~1.54) | .45 | 1.13(0.88~1.45) | .62 |  |  | 0.64(0.37~1.12) | .43 | 0.55(0.31~0.97) | .29 |  |
| [5., INF) | 2.41(1.89~3.06) | <.001 | 2.18(1.71~2.79) | <.001 |  |  | 2.66(1.78~3.98) | .02 | 2.5(1.64~3.79) | .03 |  |
|  |  |  |  |  |  |  |  |  |  |  |  |
| Numerical BSA | 0.91(0.53~1.58) | .87 | 0.94(0.53~1.67) | .91 | 0.50 |  | 2.76(1.03~7.41) | .30 | 3.39(1.21~9.45) | .23 | 0.54 |
| Categorized BSA | 1(0.92~1.08) | .98 | 1(0.92~1.08) | .96 | 0.50 |  | 1.14(1~1.3) | .30 | 1.16(1.01~1.33) | .27 | 0.55 |
| <1.54 | 1 |  | 1 |  |  |  | 1 |  | 1 |  |  |
| [1.54,1.62) | 1.02(0.85~1.22) | .91 | 1.04(0.86~1.25) | .84 |  |  | 1.58(1.16~2.17) | .14 | 1.72(1.24~2.39) | .10 |  |
| [1.62,1.69) | 1.09(0.86~1.38) | .73 | 1.09(0.85~1.4) | .72 |  |  | 1.45(0.94~2.23) | .40 | 1.5(0.95~2.36) | .38 |  |
| [1.69,1.77) | 0.96(0.67~1.38) | .92 | 0.9(0.62~1.31) | .78 |  |  | 1.42(0.76~2.63) | .57 | 1.49(0.79~2.82) | .53 |  |
| [1.77,INF) | 0.82(0.44~1.5) | .74 | 0.87(0.47~1.61) | .82 |  |  | 1.21(0.43~3.39) | .85 | 1.26(0.44~3.66) | .83 |  |

*LVEDD, left ventricular end-diastolic diameter . BSA, body surface area. bLVEDD, LVEDD divided by BSA. Age, gender, smoking within two weeks before surgery, diabetes, hypertension, hyperlipidemia, last test of serum creatinine before surgery, last test of serum total cholesterol before surgery, last test of serum low-density lipoprotein before surgery, last test of blood glucose before surgery, use of CPB, preoperative eGFR, and previous cerebrovascular events were used for the multivariate regression. bLVEDD was categorized into 4 groups based on a weight of tree-like segmentation binning.

AUC: Area under the curve.

bLVEDD: body surface area weighted left ventricular end-diastolic diameter.

BSA: body surface area.

LVEDD: left ventricular end-diastolic diameter.
